# Tailoring drug release rates in hydrogel-based therapeutic delivery applications using graphene oxide

**DOI:** 10.1098/rsif.2017.0949

**Published:** 2018-02-14

**Authors:** T. M. Puvirajesinghe, Z. L. Zhi, R. V. Craster, S. Guenneau

**Affiliations:** 1 Centre de Recherche en Cancérologie de Marseille, CRCM, Aix Marseille University, CNRS, INSERM, Institut Paoli-Calmettes, Marseille, France; 2 Diabetes Research Group, King's College London Faculty of Life Sciences and Medicine, Guy's Hospital Campus, London, UK; 3 Department of Mathematics, Imperial College London, London, UK; 4 CNRS-Imperial “Abraham de Moivre” Unité Mixte Internationale, London, UK; 5 Aix Marseille Univ, CNRS, Centrale Marseille, Institut Fresnel, Marseille, France

**Keywords:** mass diffusion, graphene oxide, drug delivery media, biocompatibility, effective model

## Abstract

Graphene oxide (GO) is increasingly used for controlling mass diffusion in hydrogel-based drug delivery applications. On the macro-scale, the density of GO in the hydrogel is a critical parameter for modulating drug release. Here, we investigate the diffusion of a peptide drug through a network of GO membranes and GO-embedded hydrogels, modelled as porous matrices resembling both laminated and ‘house of cards’ structures. Our experiments use a therapeutic peptide and show a tunable nonlinear dependence of the peptide concentration upon time. We establish models using numerical simulations with a diffusion equation accounting for the photo-thermal degradation of fluorophores and an effective percolation model to simulate the experimental data. The modelling yields an interpretation of the control of drug diffusion through GO membranes, which is extended to the diffusion of the peptide in GO-embedded agarose hydrogels. Varying the density of micron-sized GO flakes allows for fine control of the drug diffusion. We further show that both GO density and size influence the drug release rate. The ability to tune the density of hydrogel-like GO membranes to control drug release rates has exciting implications to offer guidelines for tailoring drug release rates in hydrogel-based therapeutic delivery applications.

## 1. Introduction

Biocompatible graphene oxide (GO) is increasingly used in hydrogels to chemically immobilize therapeutic agents, or to modify mechanical and thermal properties. GO can provide several therapeutic advantages such as the ability to carry bioactive molecules into hydrogels [1], as well as providing structural properties leading to increased drug-loading efficiency [2]. GO-embedded hydrogels can potentially enhance the retardation of kidney filtration with a corresponding increase in plasma half-life of therapeutic peptide and protein drugs [3]. Clinical advantages include fewer injections for patients and reduced side effects in patients.

GO is an oxidized form of graphene, which has a single-layer honeycomb lattice of carbon atoms consisting of two interlocking triangular lattices, and it has been extensively studied in various physical and chemical contexts [4,5]. The chemical structure of GO consists of epoxide and hydroxyl species in undefected carbon regions, while carboxyl, carbonyl and phenol groups are found at the edges of the carbon sheets [6]. For this reason the GO structure has been used to cross-link a variety of drugs for treating a variety of different diseases [7,8]. GO has also been shown to be able to conjugate onto polymers, thus making them amenable to scaffold structures [9]. The density of GO itself in a hydrogel can determine the rate of release and provide sustained release of a therapeutic peptide drug. Though initial studies showed that GO at high concentrations could have a dose-dependent toxicity due to the activation of oxidative stress pathways [10], it has now been shown, using *in vitro* and *in vivo* studies in a variety of cell lines, that surface modification of GO using poly(acrylic acid)-functionalization or PEGylation methods can render GO structure biocompatible [8,11,12].

Water-soluble GO has also been used for controlling diffusion of molecules through a hydrogel-like membrane. For example, GO underpinned by diffusion and percolation models has been studied for filtration applications of water molecules [13]. It has also been described as a molecular sieve allowing the permeation of ions of a certain hydrated radius [14]. Moreover, GO was used in combination with other composite materials to cross-link drugs to control drug release within the network of hydrogels [15–17]. In this model, it has been shown that the particle size of the mobile phase has an effect on mass diffusion processes within agarose gels, which involve electrostatic and other interactions, as well as a percolation effect, within fractal networks of pores [18]. Inspired by all these works, we investigate here, both theoretically and experimentally, a path towards control and delay of drug release within GO membranes and GO-embedded hydrogel networks.

One parameter that is rarely characterized is the density of the GO in hydrogel-like matrices. This can be partly blamed on the fact that suitable tools, including fluorescence recovery after photobleaching (FRAP) [19] and fluorescence correlation spectroscopy (FCS) [20], are sophisticated techniques requiring specialized and expensive equipment or expertise for data acquisition and analysis. We have side-stepped these techniques by designing a novel, yet straightforward method to measure mass diffusion based on the use of commercially available microwell inserts. We apply an effective medium approach providing crucial advantages over the other diffusion techniques, including the requirement of a basic laboratory plate reader and involving little post-experiment data analysis. Additionally, the simple method that we describe allows the simultaneous measurement of multiple conditions where the number of samples is only limited by the number of wells in the culture plate and microwell inserts. Using this method, we study how the GO density in membranes and hydrogel structures impedes the diffusion and the rate of retardation of an anti-cancer peptide drug.

We augment our experimental studies, demonstrating the control of the drug release properties of GO hydrogels, by applying an effective (two-scale homogenization) model to laminated and ‘house of cards’ configurations, to describe the percolation of the model peptide in these structures [21,22]. Using numerical stimulations, the density of GO in hydrogels can be used as a parameter to predict and optimize the rate of diffusion of the therapeutic peptide drug for controlled release. Using principles of condensed matter theory and effective medium approaches [23], one can fine-tune the drug release properties of GO hydrogels by calculating the overall GO composition (e.g. the laminated configuration leads to stronger effective anisotropic diffusivity than ‘house of cards’, and consequently to enhanced retardation effects). Our modelling method also accounts for photobleaching in the medium, which allows us to achieve a close fit between experimental data and modelling estimates, providing more accurate results for predicting the drug release rates under various conditions.

## 2. Methods

### 2.1. Peptide synthesis and diffusion experiments

The peptides used in this study were synthesized by GenScript (Hong Kong, China), according to a previously reported amino acid sequence (KL**L**LK**L**L**KK**LLK**L**LKKK, where bold characters indicate D-amino acids) [24], with conjugation of fluorescein isothiocyanate (FITC) to the N-terminal of the peptide. A fluorescence plate reader (FLUOstar Optima, BMG Labtech) (East Sussex, UK) was used to carry out the mass diffusion measurements, using filter sets appropriate for measuring FITC samples. GO in membranes was represented as volume density (μg mm^−3^). Cell culture membrane inserts with a porosity of 0.4 μm were used to study the diffusion rate of the fluorescently labelled peptide. The GO (4 mg ml^−1^ GO dispersion in water) (Sigma-Aldrich, France) was used throughout (unless otherwise specified) and deposited onto the membrane and dried by evaporation under exposure to high heat.

GO-embedded hydrogels were prepared using standard agarose (molecular biology grade) (Eurobio, France). An aqueous solution containing 2.5 wt% agarose in 2 ml of water is heated to 90°C until a clear solution is obtained. A range of different volumes of graphene suspension is added to the agarose solution with stirring. A 0.1 ml aliquot of the resulting mixture is then added in microwell inserts and the gels are allowed to solidify for 1–2 h under ambient conditions.

Diffusion measurements are carried out using 0.6 ml of 0.1 M 4-(2-hydroxyethyl)-1-piperazineethanesulfonic acid (HEPES) buffer (pH 7.40) added to the lower wells of a microwell plate and the fluorescent peptide (peptide-FITC, 0.33 mg ml^−1^ in 0.1 M HEPES) is added into the insert. A fluorescent plate reader is used to monitor the rate of drug diffusion by measuring the accumulated fluorescence intensity–time course from the lower chamber. The fluorescence measurement (bottom read) is initiated immediately under ambient conditions and monitored for several hours with a measurement cycle of 3 min. Each value is representative of an average measurement of 24 individual readings taken from the circumference of each well of the plate. The concentration of the peptide in each of the microwells is determined according to the measured fluorescence intensity using a standard curve (electronic supplementary material, figure S1).

## 3. Results and discussion

**Figure 1.**
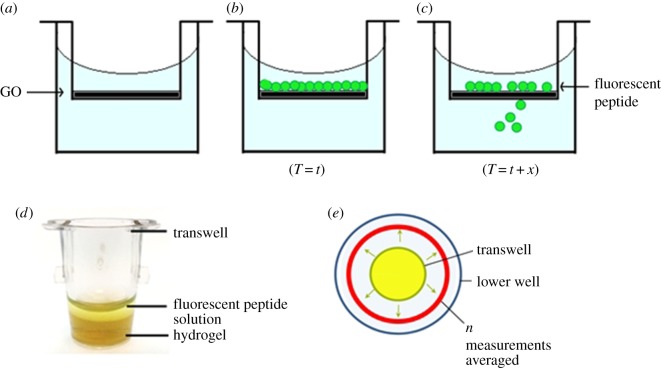
Experimental set-up for the measurement of the rate of diffusion of the fluorescent anti-cancer lytic peptide. (*a*) Schematic representation of graphene oxide (GO) deposited onto the polyethylene terephthalate (PET) membrane. (*b*) A fluorescent peptide solution is added to the Transwell insert at the beginning of the experiment (*T* = *t*). (*c*) At increasing time points during the experiment (*T* = *t* + *x*), the concentration of fluorescent peptide in the lower culture well chamber is measured. (*d*) Image showing the Transwell insert with the GO membrane or GO-embedded agarose hydrogel deposited onto the PET membrane. (*e*) Bottom-up measurements were taken using a fluorescence plate reader. A minimum of 24 individual fluorescence measurements were taken from the entire circumference of the lower well for each time point for every condition of the experiment. An average of these measurements was calculated and plotted for each time point. (Online version in colour.)

To experimentally investigate the effect of the GO density in the membrane and hydrogel matrix on the rate of diffusion of the drug molecule, we designed a simple set-up using a commercially available microwell plate, coupled with Transwell inserts and a plate reader. A previously characterized cationic anti-cancer lytic peptide was used, whose mechanism of action is based on the disintegration of the cell membrane, leading to cell death. Incubation of human breast basal epithelial cancer cells (MDA-MB-231) with different concentrations of the peptide showed previously a dose-dependent reduction in cell proliferation [24]. Employing the FITC-labelled peptide enables monitoring the presence of the peptide in diffusion wells using the microwell plate reader (electronic supplementary material, figure S1). To study the diffusion rate of the fluorescently labelled peptide, we used cell culture membrane inserts made of polyethylene membrane with the GO membrane or GO-doped hydrogel deposited onto the top of the membrane. The rate of drug diffusion was analysed by measuring the accumulated fluorescence signal (and equivalent peptide concentration) from the lower chamber of the microwells (figure 1*a*,*b*,*c* and *d*,*e*). Measurements carried out on different days using identical sample conditions showed good reproducibility and repeatability (electronic supplementary material, figure S2*a*–*c*). This shows that the curves representing the concentration of the released peptide in a function of time are the same for the measurements carried out on different days. Linear regression analysis showed that there is a linear positive regression relationship between measurements carried out on different days (*r*^2^ = 0.9981), which was highly significant (*p*-value < 0.0001), as calculated using the Prism v5.03 software. Therefore, we show that our methodology has high repeatability.

**Figure 2.**
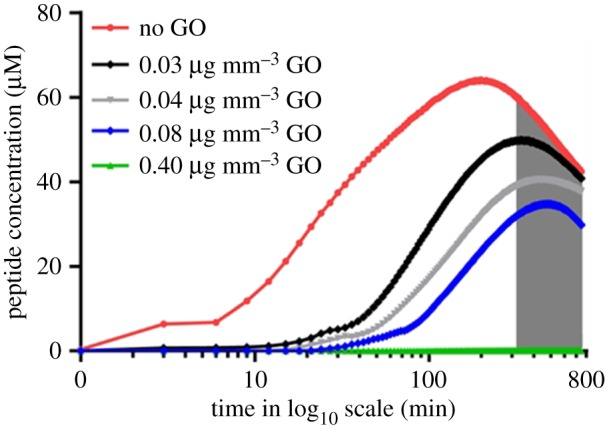
Varying the density of the GO membranes resulted in the change of the rate of diffusion of the anti-cancer lytic peptide. GO dispersion in water is prepared at different densities and dried onto translucent cell culture inserts, with a porosity of 0.4 μm. The concentration of the fluorescent peptide in the lower culture well chamber is measured and plotted against time. The period of time indicated by the grey-shaded region corresponds to the effect of photobleaching. (Online version in colour.)

The diffusion curves of the peptide (figure 2 and electronic supplementary material, figure S3) indicate that the magnitude of GO density in the membrane has a major effect on the rate of mass transport. Depositing increasing densities/quantities of GO in the membrane leads to a gradual decrease in the time taken for the peptide to reach half of its maximal intensity (*t*_1/2_). Therefore, the density of GO in the membrane determines the rate of retardation of mass transport. Increasing the GO membrane density retards, and eventually halts, the rate of mass transport (see electronic supplementary material, figure S3). In comparison to the absence of GO in the membrane, including and increasing the GO density to 0.03, 0.04, 0.08 and 0.4 µg mm^−3^ retards the peptide drug by five-, six-, sevenfold and completely, respectively. This suggests that when the density of the GO membrane is defined, it can be used as an important factor in delaying release of a therapeutic agent. The reduction of release rate of the therapeutic peptide upon increasing GO density hydrogels using this new method is reproducible (electronic supplementary material, figure S4).

**Figure 3.**
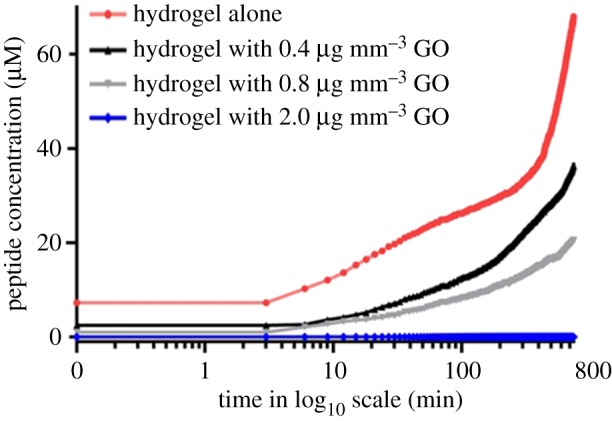
Increasing the density of the GO in agarose hydrogels resulted in the decrease of the diffusion rate of the anti-cancer lytic peptide. Different densities of GO were embedded into a 2.5 wt%/vol agarose in translucent cell culture inserts. The concentration of the fluorescent peptide in the lower culture well chamber was measured and plotted against time. (Online version in colour.)

To test the effect of GO density in a therapeutic hydrogel context, GO-doped hydrogel of agarose is used, with the concentration of fluorescent peptide kept constant. This shows that the diffusion of the fluorescence peptide through the gel barrier is significantly slower (figure 3 and electronic supplementary material, figure S5) when compared with the conditions without the hydrogel (figure 2), by approximately half. Doping of GO with an average diameter of 1 µm into the hydrogel at varying volume densities (0.4–2 µg mm^−3^) showed that a higher concentration of GO in the membrane is able to implement more reductions in the rate of diffusion of the therapeutic peptide. In particular, incorporating GO into a hydrogel to a density of 2 µg mm^−3^ results in the complete retardation in peptide diffusion within the studied period (13 h). The peptide that we used in our manuscript was fully characterized by the company where it was purchased. The peptide sequence is the following KL{*D*-L}LK{*D*-L}L{*D*-L}{*D*-L}LLK{*D*-L}LKKKC with an FITC modification occurring at the N-terminal. The peptide was characterized to have a high HPLC purity (of more than 95%) and a molecular weight measured by mass spectrometry analysis of 2667.51 Da (electronic supplementary material, data 5). In a separate study, the data from Joshi *et al*. [14] show that when a GO membrane is immersed in an ionic solution, hydration increases the GO spacing to approximately 0.9 nm. Unlike Joshi *et al*., we do not use vacuum filtration for the preparation of the GO membrane. However at higher GO membrane densities, when a more homogeneous GO membrane is attained, peptide transport does not occur and so our result is in good agreement with what was found previously by Joshi *et al*. [14].

The same experiments were then carried out using GO with a smaller average size (approx. 90 nm) (Nano Graphene Oxide Solution, Graphene Supermarket, https://graphene-supermarket.com/) included into the hydrogel. High densities of nanosized GO were able to reduce the rate of diffusion of the peptide drug (electronic supplementary material, figure S7). Compared to the absence of the GO membrane, the time taken for half the maximum amount of peptide to pass through the membrane is longer (111 versus 93 min, GO nanoparticle-containing hydrogel compared to hydrogel alone). However, hydrogels containing high densities of GO at the same time point release a third less peptide drug (i.e. *t*_1/2_ = 150 min). It should be noted that lower densities of GO have much less effect on the diffusion. Now that the predominant effect of GO density in GO-doped hydrogels is clear, it is important to be able to interpret the behaviour using numerical modelling in order to be able to predict the effect of drug release retardation.

To be able to build predictive tools to analyse the behaviour of different densities of GO, it is important to gain more information on the structural effects behind why GO density is able to assert a retardation effect on the diffusion of a therapeutic peptide. For this, transmission electron microscopy (TEM) is used to study the structural characteristics of GO density. Low GO densities showed isolation of GO structures (electronic supplementary material, figure S8*a*). However, when the density of the GO in the membrane increases, the TEM images show that the heterogeneous GO structures have the tendency to aggregate, causing overlap of GO structures (electronic supplementary material, figure S8*b*). Upon reaching a higher density of GO, complete overlap is observed, which leads to superimposition of particles, as evidenced by an overall dark grey image, shown in electronic supplementary material, figure S8*c*, thus highlighting the accumulation effects of different concentrations of GO. From the TEM images it can be noted that GO flakes share similar features to clay including the heterogeneity in the size, explaining the use of GO/clay composites [25]. GO has a unique flake-like structure, thus able to act differently when present in different densities. Increasing the density of GO in membranes may alter the percolation of molecules through an aggregated structure. In this study, we showed that the density of GO in a membrane or hydrogel is an important parameter in determining the rate of drug release. The TEM experimental data of the GO showed that GO flakes are well dispersed at low densities. However, at intermediate densities, the GO flakes tend to stack together, thus leaving empty regions in other areas. This is probably due to hydrogen bonding of water molecules in between the GO flakes. The experimental techniques devised here to study the effect of varying GO membrane density on the rate of diffusion of the therapeutic peptide are the first description in this context of a simple method to study the diffusion. Additionally, multiple conditions can be simultaneously measured using the set-up, allowing higher throughput characterization of the drug diffusion. The data obtained from the experimental analysis support the fact that the GO density in the membrane can be used to vary the rate of diffusion of therapeutic agents. The similar experiments using GO-embedded hydrogels also showed that GO membranes can provide prolonged drug release rates for the sustained drug delivery of therapeutic agents.

### 3.1. Using numerical modelling to predict drug release by varying the density of graphene oxide membranes

**Figure 4.**
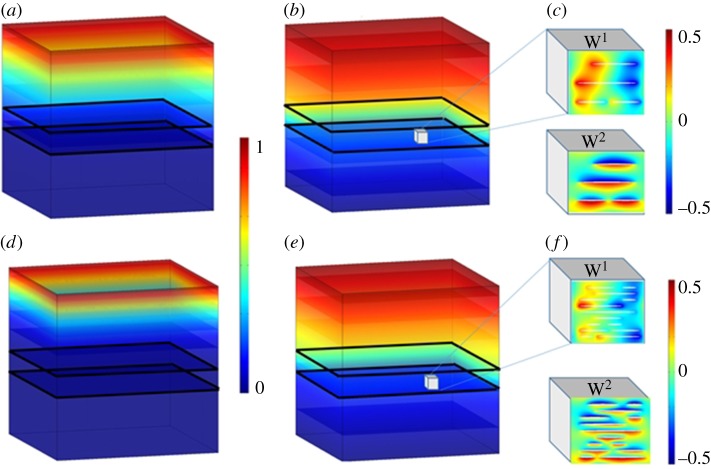
Field maps of concentration from finite-element simulations for the effective diffusion rate. (*a*,*b*,*d*,*e*) A macroscopic cubical domain of side length 20 mm with no flux boundary conditions on the left side, right side and at the bottom; an imposed concentration on the top and GO flakes (diffusivity 5500 cm^2^ s^−1^) in a water-based medium (diffusivity 1.5 × 10^−5^ cm^2^ s^−1^) overlying a porous polyethylene terephthalate (PET) membrane described by a slab 3 mm in thickness with an effective anisotropic diffusivity *D*_eff_. Concentration of the peptide at time steps *t* = 1 min (*a*,*d*) and *t* = 200 min (*b*,*e*). Peptide diffusion on low (0.03 µg mm^−3^) (*a*,*b*) and intermediate (0.08 µg mm^−3^) (*d*,*e*) density of GO flakes (in units of ng mm^−3^). Linear colour scale ranges from vanishing (dark blue) to high (red) concentration normalized to 1. (*c*,*f*) A microscopic cubical domain of typical side length *η* small compared to X (1 µm or less) with periodic boundary conditions on opposite sides for low (*c*) and high (*f*) density of GO flakes displays periodic potentials W^1^ and W^2^ associated with effective anisotropy *D*_eff_. Linear colour scale ranges from negative (dark blue) to positive (red) values of W^1^ and W^2^ normalized to 1. (Online version in colour.)

**Figure 5.**
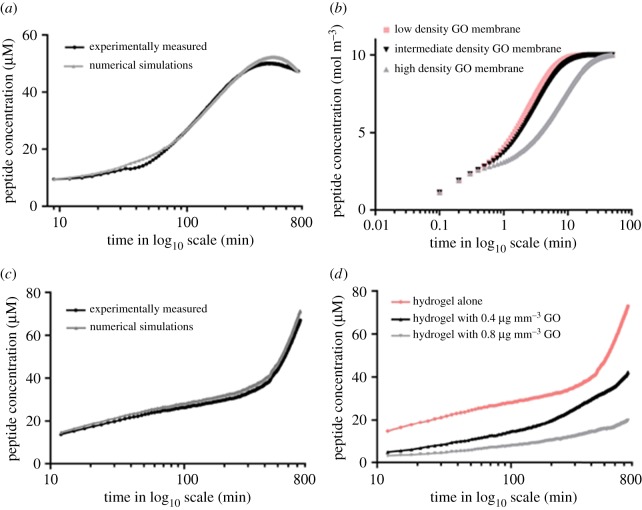
Curves of the peptide concentration from finite-element simulations for the effective diffusion rate. (*a*) Diffusion model for photobleaching for figure 2: the variation over time (min) of the peptide concentration (μM) for a density of GO of 0.01 µg mm^−3^ observed experimentally (black line) is compared against numerical simulations carried out using finite-element computations (grey line) for a diffusion model with effective parameters as given by equation (3.2). The value of parameter *α*_eff_ (alpha effective) in equation (3.2) is approximately 10^−8^ s^−1^. Note that the values during 1–10 min were excluded from the plot due to difficulties in matching the experimental and numerical curves. (*b*) Numerical simulations showing the variation in the concentration of the peptide with time for low (0.05), intermediate (0.1) and high (0.5) densities of GO flakes (in units of μg mm^−3^). (*c*) Diffusion model without photobleaching for figure 4: same as (*a*) for GO in hydrogel. (*d*) Numerical simulations showing the same as (*b*) for hydrogels with and without GO. Normalization was applied between numerically computed and experimentally measured peptide concentrations at the initial time step in order to compare similar data. Diffusion coefficient for the molecule is based on an agarose hydrogel [18]. (Online version in colour.)

The numerical simulations presented in this study are the first demonstration of the use of commercial finite element software COMSOL Multiphysics (figures 4 and 5) for the determination of the diffusion rate of a therapeutic peptide. Our results show that we are able to clearly reproduce most of the features of the experimental data (figures 2 and 3). In this way, we are now able to predict the outcomes of drug release from GO membranes and GO hydrogels by considering membranes and hydrogels of varying GO densities.

We applied an effective model for porous media to obtain the numerical results (figures 4 and 5). The effective and ‘free’ diffusivities are usually related according to the equation *D*_eff_ = *Dɛ*/*τ*, where *ɛ* is the porosity of the structure and *τ* is the tortuosity, which is a measure of the actual length per unit effective length a molecule has to diffuse in a porous structure. Porosity of the modelled structure was calculated by considering the ratio, we considered the ratio of the perforations to the total computational area; the concentration then obeys Fick's equation [26]:3.1
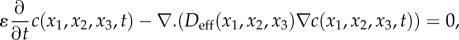
where 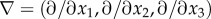 denotes the gradient with partial derivatives with respect to space variables *x_i_*. Tortuosity is usually expressed as a power of the porosity [27], therefore the effective diffusivity varies as *D*_eff_ = *Dɛ^p^*.

We note that applying Fick's equation simply leads to an increase in diffusivity, reaching a plateau at longer time points, which corresponds to the rate of diffusion of an anti-cancer lytic peptide in GO hydrogels (figure 5*c*,*d*), but does not explain the relative decrease observed in concentration of the same anti-cancer drug when it diffuses through a GO membrane without hydrogel (figure 5*a*,*b*).

Our interpretation is that the physical system suffers degradation due to photobleaching at longer time points. We incorporated this factor of decay by using an additional term: 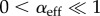 which is the rate of photobleaching [28–30]:3.2
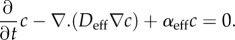


We verified that the concentration versus time followed the same trend in figure 2 and figure 5*a* for a value of parameter *α*_eff_ in equation (3.2), which is approximately 10^−8^ s^−1^. Regarding figure 3 and figure 5*c*,*d*, which display a monotonically increasing solution to the diffusion equation, the parameter *α*_eff_ in equation (3.2) is zero, because no photobleaching is observed. This can be attributed to the fact the peptide is retarded in hydrogels and photobleaching effects are only evident several hours after a plateau in intensity has been reached; as the peptide in hydrogels took 13 h to reach this maximal fluorescence intensity, the effects of photobleaching cannot be measured.

In addition, this experimentally observed photobleaching was incorporated into the numerical modelling. The effect of photobleaching is evident in the experiments using micron-sized GO membranes, which, unlike in membranes, have higher peptide diffusion rates during the experimental time points. Hydrogel experiments would have to be carried out for much longer time periods for the effects of photobleaching to be investigated in GO hydrogel. This shows that, after a period of time, the peptide concentration decreases in GO membranes (figure 2), unlike the monotonically increasing concentration in GO hydrogels (figure 3). Photobleaching is accounted for in figure 2 by adding an *ad hoc* term to the diffusion equation (figure 5*a–d*), which is unnecessary for figure 4 as numerically checked in figure 5*c*,*d*, wherein the parameter accounting for photobleaching is turned off. Therefore, we are able to fit the experimental data to the modelling data in both GO membranes and GO hydrogels. It should also be noted that, in the course of our experimental study of GO hydrogels, we observed that varying the density of nanosized GO in hydrogels does not further delay the rate of diffusion of an anti-cancer lytic peptide (electronic supplementary material, figure S6), compared with GO hydrogels with the same density of (micrometre-sized) GO (figure 3 and electronic supplementary material, figure S3).

These experiments could be probably interpreted, as described in [13], using an additional driving mechanism that can be attributed to the interaction between monolayer hydrogel (water in [6]) and the graphene walls similarly to a theoretical experiment involving a 2D channel with a left and a right reservoir and an effective capillary pressure **P**. When the channel is fully filled, and an additional force is applied to all oxygen atoms in the direction against the flow, this mimics a gravitational force and can directly be translated into an extra-capillary pressure **P** in the slit. It was found that if **P** exceeded ≈500 bars [13], water molecules in the channel were pulled back to the left reservoir, and the 2D capillary started to dry out. The value of **P** found in their 1 nm-sized slit is in qualitative agreement with the classical estimates using the van der Waals interaction [31,32] between water and graphite [13]. In the present work, the van der Waals interaction would be an adverse effect to delayed drug diffusion in hydrogels with nanosized particles.

However, equation (3.2) does not account for effective anisotropic features that one would expect for a medium filled with extremely elongated inclusions like GO flakes: indeed, the percolation behaviour of anisotropic particles can be subtle and often counter-intuitive as noted in [33]. So we further compare our effective model against a homogenized Fick's equation obtained from a two-scale asymptotic approach for a porous medium consisting of a background with diffusivity *D*_0_ surrounding inclusions that have high diffusivity 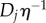 where 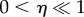 and 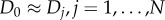. Note that this model assumes certain periodicity in the medium, i.e. the set of inclusions 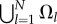 should repeat periodically with periodicity *η* in all three space dimensions, so we can define a periodic cell 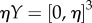. This means one considers a medium with a large number of periodic cells themselves filled with a large number of inclusions. For more details on this asymptotic approach, which assumes two separate scales with macroscopic (so-called slow) variables 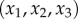 and microscopic (fast) variables 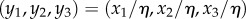, one can find a vast amount of literature, but the most relevant references are [34,35]. Expanding the concentration as a Taylor series 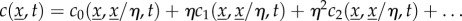 and rescaling the gradient as 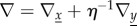, a hierarchy of partial differential equations is obtained. By collecting terms, integrating quantities over the periodic cell, and invoking the divergence theorem leads to (see also electronic supplementary materials)3.3
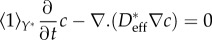
3.4
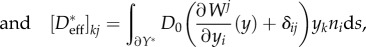
where 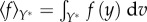 and 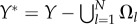 is the volume of the periodic cell *Y* excluding all inclusions and the unknown functions *W^j^* are solutions of annex problems on a periodic cell Y*3.5
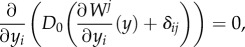
with 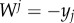 on the boundary of each inclusion i.e. on 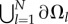 and 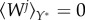.

Solving the annex problem equation (3.5) for a periodic cell shown in figure 4, which we take to be representative of the GO membrane, we compute an effective diffusivity according to equation (3.4) with diagonal entries 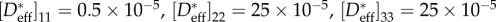 and vanishing off-diagonal elements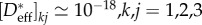. However, we found this gives curves with not entirely satisfactory agreement with experimental results, so we once again resorted to the percolation model, which now takes the form (with effective anisotropy, porosity and tortuosity encapsulated, with the values *ɛ* = 1/2 and *p* = 3/2 assumed to be reasonable from [27]):3.6
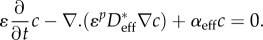


For the sake of consistency (see electronic supplementary material, figures S9 and S10), we compare this high-contrast model with the numerical solution of the classical annex problem for a multiphase medium, where Y* is replaced by Y in equation (3.3)–(3.5) and we now assume continuity of potential *W^j^* and its flux 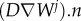 across the boundary of each inclusion where *n* is the inward pointing unit normal on 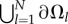. Our numerical results lead to the same effective diffusivity for the high-contrast and multiphase annex problems. We further checked (see electronic supplementary material, figure S11) that a ‘house of cards’ configuration for GO flakes leads to an effective diffusivity with reduced anisotropy in comparison with the laminated configuration, and consequently less retardation in drug delivery. Finally, in the course of the numerical simulations, we needed to calibrate space and timescales with canonical examples of GO flakes models (see electronic supplementary material, figure S12).

In conclusion to this modelling section, the experimental data provided strong evidence to support our suggestion that the density of the GO membrane can be used to modify the rate of release of therapeutic drugs. Both laminated and ‘house of cards’ models are used to model GO in numerical and analytical simulations due to the resemblance of such assemblies to those of GO structures. Our numerical investigations based on the effective equation (3.6) demonstrate that the former, laminated, configurations exhibit strong effective diffusivity that is more conducive for diffusion delay. Importantly, the effective model requires a large number of small (periodically assembled) cells, but it does not give any more information regarding the scaling (the small parameter *η* need only be small compared to the typical dimensions of the polyethylene terephthalate (PET) membrane, so we get the same effective diffusivity for nano- and micrometre-sized GO flakes). Thus, in our opinion, we cannot completely rule out a possible effect of van der Waals forces for nanometre-sized GO flakes, because experiments show a further diffusion delay in that case (which is not encapsulated by our homogenization model). Interestingly, it has been previously shown that a homogenization approach of Fick's equation can be used to control processes of mass diffusion for biological and engineering applications [36,37]. To assess *the fit* of the model to the experimental data, the acquired experimental data were compared with the effective mathematical models. The same equation as in [36] is used to retrieve in (figure 5*c*,*d*) the main features of the experiments shown in (figure 3). However, one notes that there is a decrease in the concentration after a certain time in (figure 2): this effect is the result of photo-thermal degradation of fluorophores. To account for this in the numerical modelling, a certain activation term *α*_eff_ is added in the diffusion equation (3.2) as described in the methods, and as a result one gets figure 5*a*,*b*, which reproduces the observed experimental effect.

It should be noted that the experimental set-up described and used in figures 1–2 avoided evaporation of aqueous solution by using a parafilm coating of the upper and lower chambers. Therefore, the dominant effects can be considered to be associated with diffusion and photobleaching, whereas convection and advection can be ruled out. The peptide molecule (the FITC part) is sensitive to photobleaching as shown in electronic supplementary material, figure S9. This shows that the fluorescent peptide which is exposed to light for 2 h results in a reduction of the overall fluorescence intensity compared to the peptide protected from light and kept and stored under the same conditions.

The results described in this manuscript have shown that the micrometre-scale and nanometre-scale GO can be incorporated into hydrogels in order to provide sustained delivery of a therapeutic peptide drug and thus avoid a ‘burst release’ of the drug and improve drug efficacy. We believe that our results show that the GO can have superior qualities over other 2D biocompatible materials such as bioceramics. Some of the advantages over these materials are that it is easily available in different forms (easier to fabricate), and it has higher tensile strength and provides a tough biocompatible material, which can be available at the micrometre and nanometre scale for incorporation into biological scaffolds. Other types of scaffolding materials such as polymer scaffolds can degrade in an uneven manner [38], but GO does not have that issue. GO is also available at a cheaper cost.

## 4. Conclusion

Given that GO has great potential to be employed in biological contexts such as hydrogel membranes, we demonstrate an additional advantage to using GO in that the delayed drug diffusion in GO membranes and hydrogels can be achieved by controlling the GO density. Varying the density of GO in the membranes means that the network structure becomes less permeable to varying degrees. We used principles of an effective percolation model to consider the movement of a peptide drug in GO membranes. For optimization of the density of GO in the membrane to achieve the desired drug diffusion rate, we employed numerical simulations which included the experimental parameters such as physical movement of the molecules and the photo-thermal degradation of fluorophores. Therefore, this type of modelling will be useful for contexts such as the prediction of the drug release properties of the hydrogels for therapeutic applications; this aids in cutting down the amount of wet-lab materials required and, by screening different criteria, this reduces the time taken for experiments. In the context of hydrogels, it is evident that diffusion rates of the peptide drug are significantly impeded with the increased density of the doped GO. However, varying both the density and the size of the GO could be the key to achieving the most efficient peptide release rates.

## Supplementary Material

Supplementary Methods, Results and Figures

## Data accessibility

Additional data and analyses are made available in the electronic supplementary material, appendix A, B and C.
