# Supplementary material for: Tailoring drug release rates in hydrogel-based therapeutic delivery applications using graphene oxide
Source: J R Soc Interface. 2018 Feb 14;15(139):20170949. doi: 10.1098/rsif.2017.0949 (PMC5832740; doi:10.1098/rsif.2017.0949)

**SUPPLEMENTARY MATERIALS**

# Tailoring drug release rates in hydrogel-based therapeutic delivery applications using graphene oxide

T. M. Puvirajesinghe*, Z.L. Zhi, R. V. Craster, and S. Guenneau.

*Corresponding author. E-mail: tania.guenneau-puvirajesinghe@inserm.fr and sebastien.guenneau@fresnel.fr

INVENTORY OF SUPPLEMENTARY INFORMATION AND DATA

Supplementary Methods

Supplementary Results

Supplementary Figures

Supplementary Figure 1

Supplementary Figure 2

Supplementary Figure 3

Supplementary Figure 4

Supplementary Figure 5

Supplementary Figure 6

Supplementary Figure 7

Supplementary Figure 8

Supplementary Figure 9

Supplementary Figure 10

Supplementary Figure 11

Supplementary Figure 12

Supplementary Methods

*Transmission electron microscopy (TEM)*

GO dispersion in water, 4 mg/mL, was diluted 100 times with water. 2 µL of the diluted solution was deposited onto an electron microscopy grid. After 20 seconds, the drop is dried with a small fragment of filter paper. A 200 kV with a Tecnai G2 (FEI, Netherlands) and Velata camera (Olympus, Japan) is used for image acquisition.

Supplementary Results

The fluorescence intensity of the therapeutic peptide has a linear positive regression relationship (r=0.8) with the peptide concentration. Therefore the concentration of the peptide can be calculated using the fluorescence intensity (**Supplementary figure** *1*). The experimental setup described in the main article is reproducible as experiments carried out using the same conditions on different days obtained the same results (**Supplementary figure** *2*). Main **figure** *2* is shown on $log_{10}$ scale with the sample data shown in the linear scale in **Supplementary figure** *3*. Main **figure** *3* graphs are shown on $log_{10}$ scale and the same data is shown with the Y coordinates plotted in the linear scale **(Supplementary figure** *4)*. The molecular weight of the therapeutic peptide is shown in **Supplementary figure** *5*. A hydrogel containing nanosized GO flakes shows that GO can be used to reduce the rate of diffusion of a peptide drug (**Supplementary figure** *6*). But the retardation effects are less potent than with micrometer sized GO flakes. Therefore both the concentration and size of GO are important parameters. The structural characteristics of GO density were analyzed using TEM. Based on the fact that GO consists of a high proportion of carbon atoms, for which the low atomic number reduces scattering of the electron beam, a good contrast and a sharp image were acquired in TEM experiments without the need of the addition of a contrast agent. The aggregation and behavior of different concentrations of GO is analysed (**Supplementary figure** *7*). The effect of photobleaching on the FITC-peptide is depicted in **Supplementary figure** *8*. **Supplementary figures** *9-12* describe the numerical modelling to predict drug release by varying the density of GO membranes.

**Supplementary Figure 1: Calibration curve of fluorescence intensity and concentration of the peptide.** Calibration curves of peptide concentration vs. fluorescence intensity; the graph shows the linear positive regression relationship (r=0.8) that was used to determine the peptide concentration.

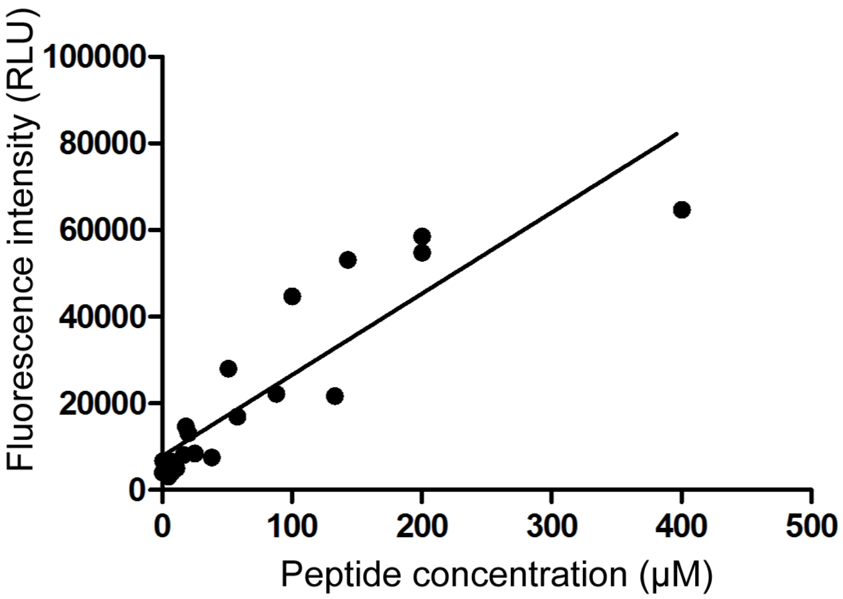

**Supplementary Figure 2: Reproducibility of diffusion rate measurements.** *(a)* As the *x* values are identical for all measurements, repeatability was assessed by plotting the *y* values from two experiments using the same experimental parameters and using linear regression analysis. *(a)* Linear regression analysis carried out for experiments using a GO membrane of 0.03 μg/mm$^{3,}$ using Prism v5.03 software. Data is displayed on a $\log_{10}$ scale. *(b)* Linear regression analysis carried out for experiments using a GO membrane of 0.04 μg/mm$^3$. *(c)* Linear regression analysis carried out for experiments using a GO membrane of 0.08 μg/mm$^3$.

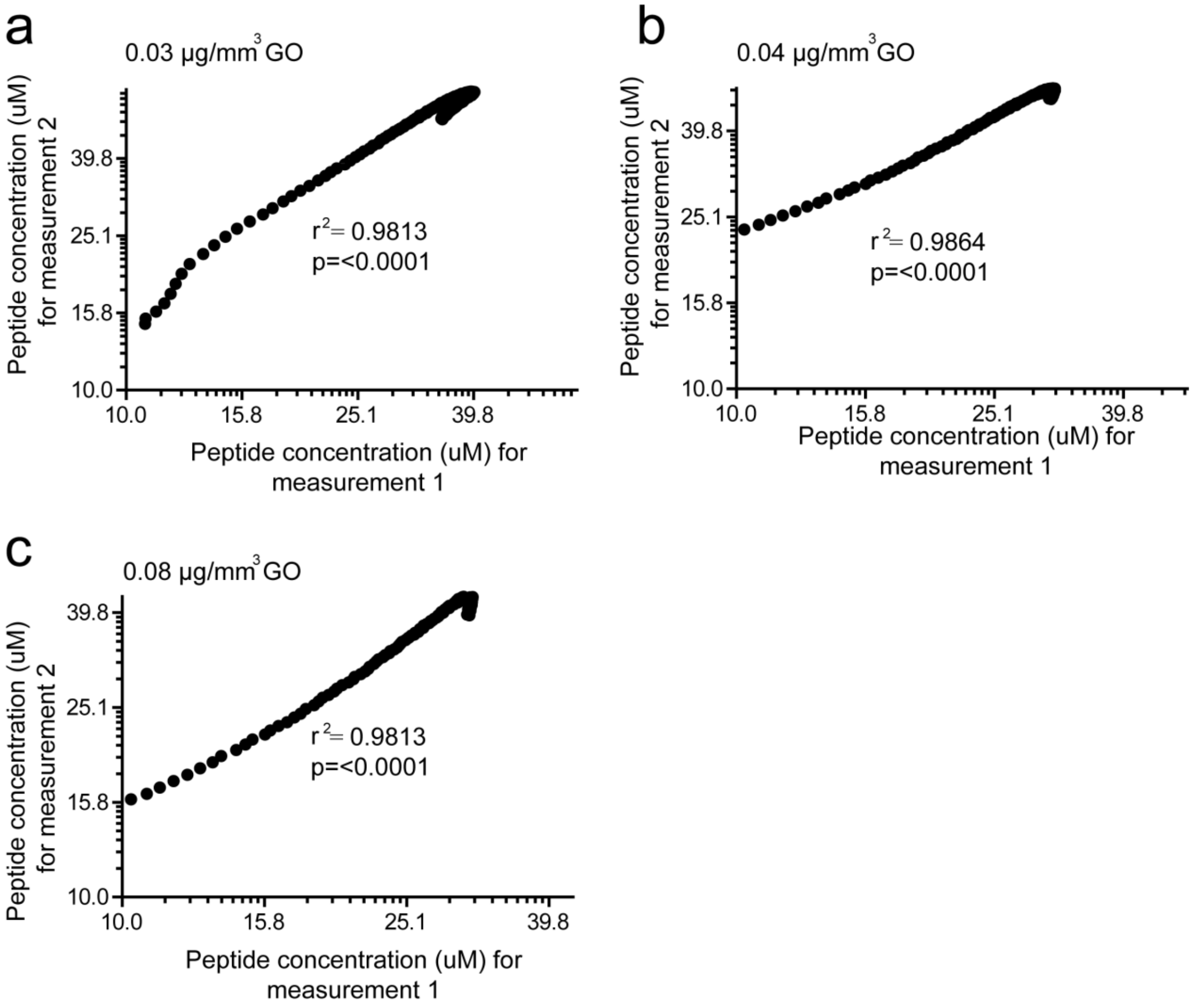

**Supplementary Figure 3: Varying the density of the GO membranes controls the rate of diffusion of an anticancer lytic peptide.** Graph is shown on a linear scale and corresponds to figure 2 of the main article (which is on $\log_{10}$ scale).

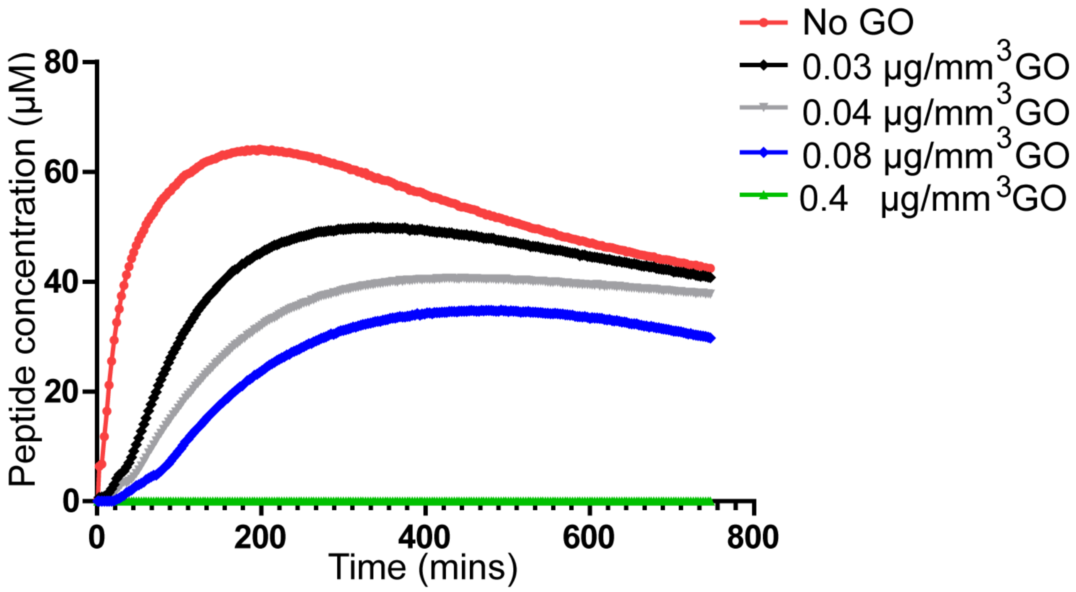

**Supplementary Figure 4: Varying the density of the GO membranes in a hydrogel controls the rate of diffusion of an anticancer lytic peptide.** Graph is shown on a linear scale and corresponds to figure 4 in the main article (which is on $\log_{10}$ scale). The gel was introduced into the translucent cell culture inserts made from PET membrane and allowed for 2 h to solidify under ambient conditions. The concentration of the fluorescent peptide in the lower culture well chamber is measured and plotted against time. Each reading is representative of an average measurement of 24 readings taken from the circumference of each well of the culture dish. Readings are taken every 3 min during a period of 13 h.

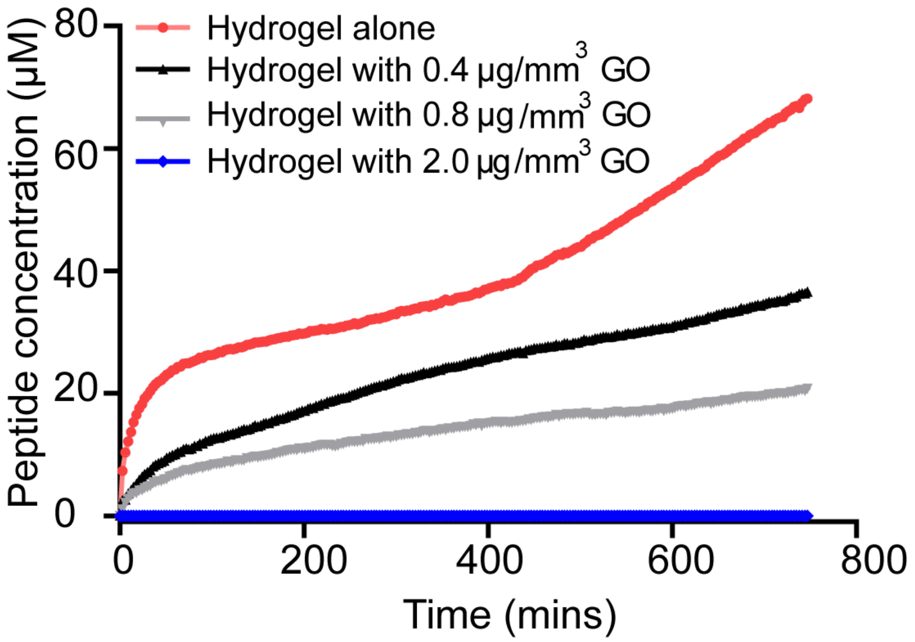

**Supplementary Figure 5: Molecular weight and purity of the peptide compound used in this study.** The peptide sequence is the following KL{*D*-L}LK{*D*-L}L{*D*-L}{*D*-L}LLK{*D*-L}LKKKC with a FITC- modification occurring at the N-terminal. HPLC analysis was used to confirm a purity of ≥95.0 %. Mass spectrometry data was used to confirm that the molecular weight of the compound is 2667.51 Da. Data provided by GenScript.

A

Sample Name : 1
Sample ID : U5140BE120-1
Pump A : 0.065% trifluoroacetic in 100% water (v/v)
Pump B : 0.05% trifluoroacetic in 100% acetonitrile (v/v)
Total Flow:1 ml/min
Wavelength:220 nm

| Time | Unit | Command | Value | Comment |
|---|---|---|---|---|
| 0.01 | Pumps | Pump B Conc. | 5 | |
| 25.00 | Pumps | Pump B Conc. | 65 | |
| 25.01 | Pumps | Pump B Conc. | 95 | |
| 31.00 | Pumps | Pump B Conc. | 95 | |
| 31.01 | Pumps | Pump B Conc. | 5 | |
| 40.00 | Pumps | Pump B Conc. | 5 | |
| 40.01 | Controller | Stop | | |

<<Column Performance>>
<Detector A>
Column : AlltimaTM C18 4.6 x 250 mm

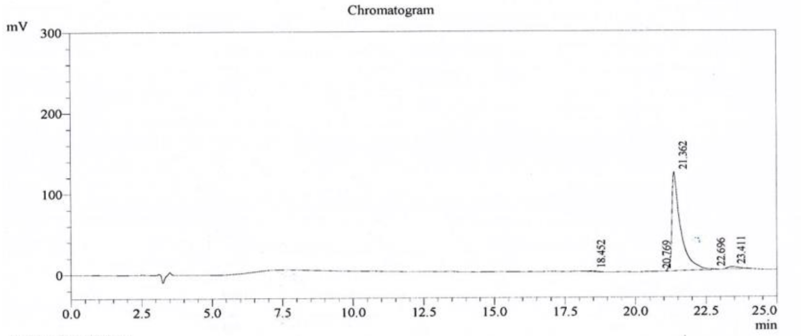


1 Det.A Ch1 / 220nm

Peak Table

Detector A Ch1 220nm

| Peak# | Ret. Time | Area | Height | Area % |
|---|---|---|---|---|
| 1 | 18.452 | 18711 | 1021 | 0.687 |
| 2 | 20.769 | 537 | 86 | 0.020 |
| 3 | 21.362 | 2615404 | 122801 | 96.060 |
| 4 | 22.696 | 8134 | 911 | 0.299 |
| 5 | 23.411 | 79892 | 2770 | 2.934 |
| Total | | 2722678 | 127589 | 100.000 |

B

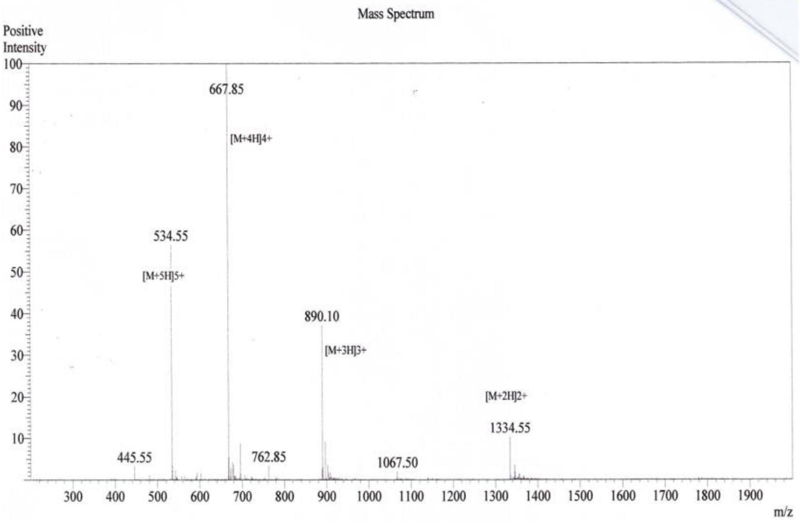


Sample Information
Acquired by : Gary
Month-Day Processed : 06/02/16
Time Processed : 10:53:11
Injection Volume : 0.2
Sample Name : 1
Sample ID : U5140BE120-1
Theoretical MW : 2667.51
Observed MW : 2667.40

Interface :ESI
Nebulizing Gas Flow :1.5L/min
CDL Temp :250℃
Block Temp :200℃

Interface Bias : +4.5 kV
Drying Gas Flow :5 L/min
T.Flow :0.2 ml/min
B.conc :50%H2O/50%MeOH

**Supplementary Figure 6: Varying the density of the nanosized GO in hydrogels controls the rate of diffusion of an anticancer lytic peptide.** Different densities of GO were added into a 2.5 wt%/vol agarose with stirring. The gel was introduced into the translucent cell culture inserts made from PET membrane and allowed for 2 h to solidify under ambient conditions. The concentration of the fluorescent peptide in the lower culture well chamber is measured and plotted against time. Each reading is representative of an average measurement of 24 readings taken from the circumference of each well of the culture dish. Readings are taken every 3 min during a period of 13 h.

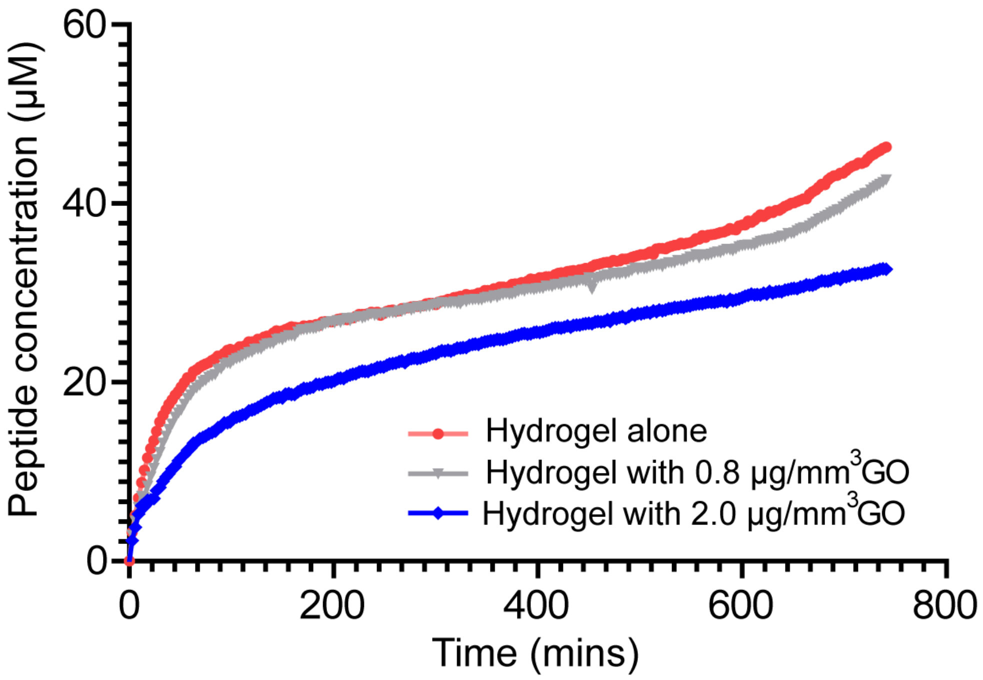

**Supplementary Figure 7: TEM images showing GO of varying densities**. Images are acquired using a Morgagni FEI 80KV Camera digital View III Olympus camera. For clarity the prominent sheets are outlined with dashed lines. (*a***)** An image of one sheet, note that the contrast is very similar to that of the background surface. (*b***)** Several sheets, each sheet is again outlined and the contrast is increased when additional sheets are superimposed. (*c*) Multiple sheets showing a sharp increase in the contrast when many GO flakes are stacked together, thereby substantially increasing the gray scale of the images. Scale bar (500 nm).

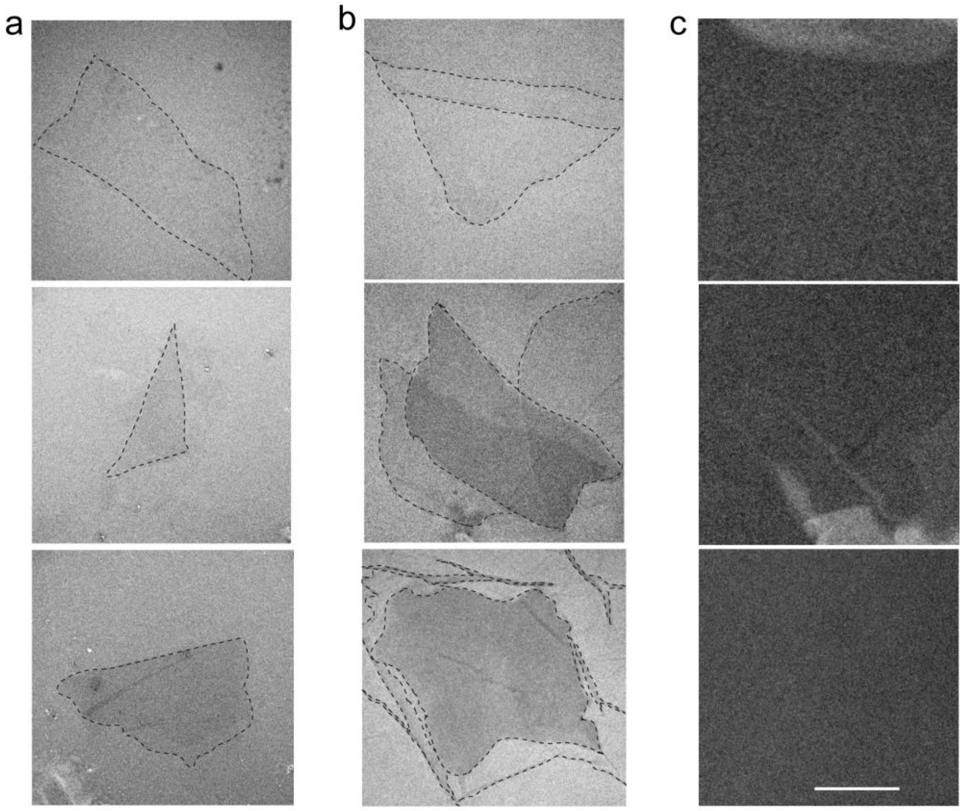

**Supplementary Figure 8: Effect of photobleaching of FITC-conjugated peptide used in the study.** Following exposure to white light for varying amounts of time, the fluorescence intensity of the FITC-conjugated peptide was measured. Initial fluorescence intensity is indicated as the dashed black line. Fluorescence of samples is expressed as reduction of percentage of fluorescence intensity compared to the florescence intensity (arbitrary units (AU) at time point 0). After 8 h of exposure a loss of 50 % percentage of the initial fluorescence is lost.

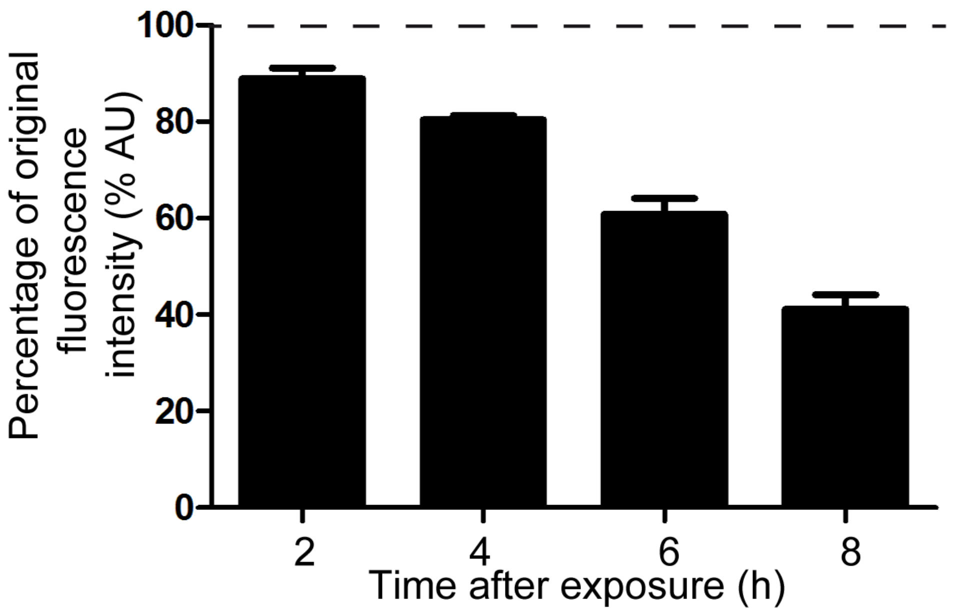

**Supplementary Numerical Results:** Using numerical modelling to predict drug release by varying the density of GO membranes.

For the sake of completeness, we give the form of the homogenized Fick's equation Eq. (1.3), its effective diffusivity Eq. (1.4) and the annex problem Eq. (1.5) in the case of multi-phase media (without assuming a high contrast in diffusivity) and integrating quantities over the periodic cell Y and invoking the divergence theorem leads to

$$<1>_Y \frac{\partial}{\partial t} c(x_1, x_2, x_3, t) - \nabla.(D^c_{eff} \nabla c(x_1, x_2, x_3, t)) = 0 \tag{1.1}$$

$$[D^c_{eff}]_{kj} = \int_{Y*} D_0 \left( \frac{\partial W_0^j}{\partial y_i}(y) + \delta_{ij} \right) dv + \sum_{l=1}^{N} \int_{\Omega_l} D_l \left( \frac{\partial W_l^j}{\partial y_i}(y) + \delta_{ij} \right) dv, i, j, k = 1,...,3 \tag{1.2}$$

Where $< f >_Y = \int_Y f(y) dv$, $y = (y_1, y_2, y_3)$ and $Y^* = Y - \bigcup_{l=1}^{N} \Omega_l$ is the volume of the periodic cell $Y = [0, a_1] \times [0, a_2] \times [0, a_3]$ excluding all inclusions $\mathrm{W}_l$ and the unknown functions $W_0^j$ and $W_l^j$ (so-called potentials), are solutions of the following annex problems on a periodic cell $Y$

$$\frac{\partial}{\partial y_i} \left( D_0 \left( \frac{\partial W_0^j}{\partial y_i}(y) + \delta_{ij} \right) \right) = 0 \tag{1.3}$$

$$\frac{\partial}{\partial y_i} \left( D_l \left( \frac{\partial W_l^j}{\partial y_i}(y) + \delta_{ij} \right) \right) = 0, l = 1,..., N \tag{1.4}$$

With continuity of the potentials

$$W_0^j = W_l^j, l = 1,..., N \tag{1.5}$$

on the boundary of each inclusion i.e. on $\bigcup_{l=1}^{N} \partial \Omega_l$ and continuity of their flux

$$D_0 \left( \frac{\partial W_0^j}{\partial y_i}(y) + \delta_{ij} \right) n_j = D_l \left( \frac{\partial W_l^j}{\partial y_i}(y) + \delta_{ij} \right) n_j \tag{1.6}$$

With a final condition ensuring uniqueness of the solutions up to an additive constant

$$\int_{Y^*} D_0 W_0^j(y) dv + \sum_{l=1}^{N} \int_{\Omega_l} D_l W_l^j(y) dv = 0 \tag{1.7}$$

Of course, if we consider some 2D periodic cell $Y = [0, a_1] \times [0, a_2]$, one only has to compute 2 unknown potentials in each homogeneous phase, and using transmission conditions Eq. (1.5) and (1.6) these can be combined in 2 potentials $W^1$ and $W^2$ defined over the entire cell $Y$.

We would like also to mention that in the limit case of a bi-layered structure i.e. when one considers an alternation of layers of diffusivities $D_0$ and $D_1$ and thicknesses $1-h$ and $h$ along variable $y_1$ so a periodic cell $Y=[0,1]\times[0,1]\times[0,1]$ where the sidelength along $y_2$ and $y_3$ has been normalized. One then solves analytically Eq. (1.3) and (1.4) by noting that $W_0^j$ and $W_1^j$ are functions of $y_1$ only:

$$\frac{d}{dy_1}\left(D_0\left(\frac{dW_0^1}{dy_1}(y_1)+1\right)\right)=0 \tag{1.8}$$

$$\frac{d}{dy_1}\left(D_1\left(\frac{dW_1^1}{dy_1}(y_1)+1\right)\right)=0 \tag{1.9}$$

With continuity Eq. (1.5) and (1.6): $W_0^1=W_1^1$ and $D_0\left(\frac{dW_0^1}{dy_1}(y_1)+1\right)=D_1\left(\frac{dW_1^1}{dy_1}(y_1)+1\right)$.

Taking into account the zero mean Eq. (1.7), we find

$$W_0^1=\frac{\mathrm{h}(\mathrm{D}_2-\mathrm{D}_1)}{hD_1+(1-h)D_2}\left(y_1-\frac{(1+h)}{2}\right) \tag{1.10}$$

$$W_1^1=\frac{(1-\mathrm{h})(\mathrm{D}_2-\mathrm{D}_1)}{hD_1+(1-h)D_2}\left(y_1-\frac{h}{2}\right) \tag{1.11}$$

Plugging Eq. (1.10) and (1.11) in Eq. (1.2), we obtain the effective tensor of diffusivity:

$$[D_{eff}^c]_{11}=\frac{D_1D_2}{hD_1+(1-h)D_2},[D_{eff}^c]_{22}=[D_{eff}^c]_{33}=hD_2+(1-h)D_1 \tag{1.12}$$

With zero off-diagonal entries.

It is interesting to note that in the limit when $D_2/\mathrm{D}_1$ goes to infinity, $[D_{eff}^c]_{11}$ tends to zero and $[D_{eff}^c]_{22}=[D_{eff}^c]_{33}$ tends to infinity, hence the effective diffusivity becomes infinity anisotropic. In this limit case, the drug delay by the GO agarose membrane would be infinite. So one needs to consider some laminated structure with interstitial space between GO flaxes so that drug can diffuse through the membrane, but intuitively the larger the anisotropy of the effective diffusivity, the longer the drug delay. In order to match the experimental data, we thus considered various configurations of laminated structures and numerically computed the corresponding effective diffusivity. But we note that this kind of inverse engineering problem

does not have a unique solution, so what we show in **Supplementary figures** *9* and *10* are simply representative configurations for our GO agarose membranes for low (0.03 μg/mm$^3$) and intermediate (0.08 μg/mm$^3$) density of GO flakes. As it turns out, we find that a 'house of cards' configuration, see **Supplementary figure** *11*, would also fit experimental data, although in this case the 'randomized' orientation of GO flakes leads to a tensor of diffusivity, which has all its entries non vanishing, and this smears down the strong anisotropy in the xy-plane, so that diffusion delay is less pronounced. We note that there is a critical scaling between spatial and time steps for the numerical simulations, and this was tested on simple GO configurations to get a grasp of the physical phenomena involved, see **Supplementary figure** *12*.

**Supplementary Figure 9: Numerical solutions $W^1$ (a) and $W^2$ (b) of annex problem Eq. (1.5):** for low concentration (0.03 µg/mm$^3$) of GO ($D_1$=5.5 x10$^3$ cm$^2$/sec) in water ($D_0$=1.5 x10$^{-5}$ cm$^2$/sec) compared against $W^1$ (c) and $W^2$ (d) for infinitely diffusive inclusions. In both case, effective diffusivity computed with Eq. (1.4) is $D_{eff} = \begin{pmatrix} 17 & 0 \\ 0 & 0.3 \end{pmatrix} 10^{-5}$ .

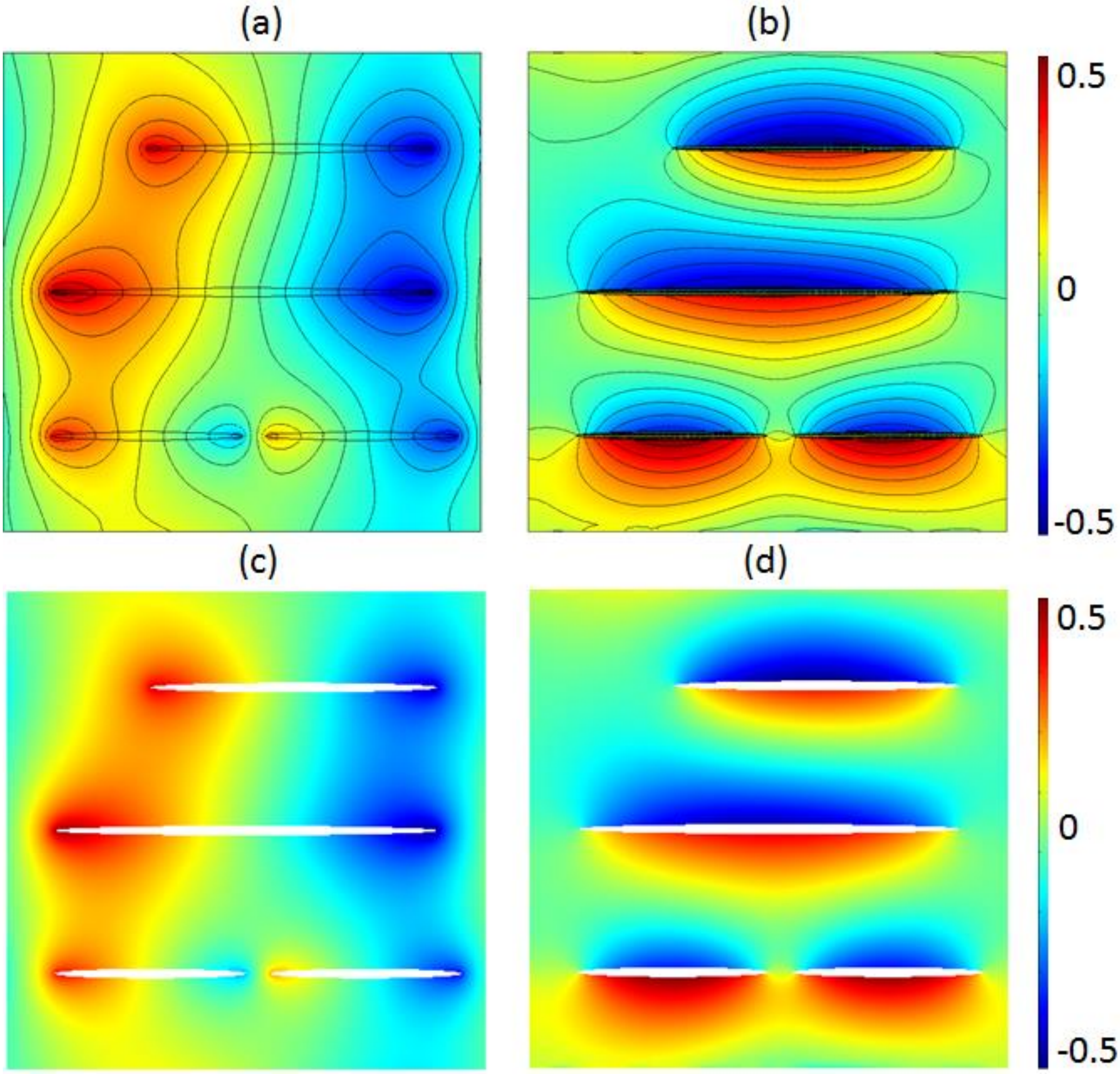

**Supplementary Figure 10: Numerical solutions $W^1$ (a) and $W^2$ (b) of annex problem Eq. (1.5) in a laminated configuration:** for intermediately high (0.08 μg/mm$^3$) concentration of GO ($D_1$=5.5 $10^3$ cm$^2$/sec) in water ($D_0$=1.5 $10^{-5}$ cm$^2$/sec) compared against $W^1$ (c) and $W^2$ (d) for infinitely diffusive inclusions. In both case, effective diffusivity computed with Eq. (1.4) is $D_{eff} = \begin{pmatrix} 25 & 0 \\ 0 & 0.2 \end{pmatrix} 10^{-5}$ .

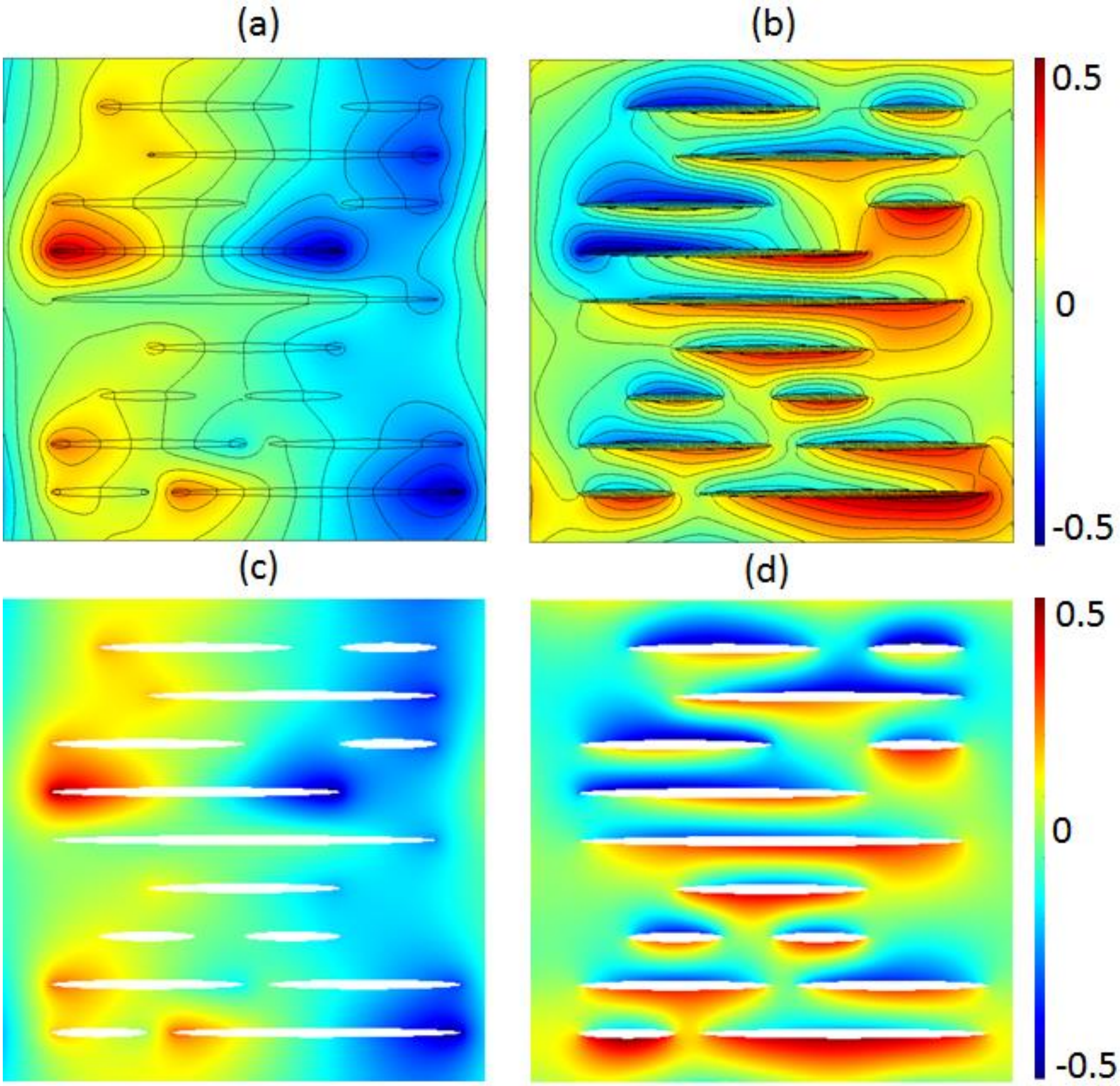

**Supplementary Figure 11: Numerical solutions $W^1$ (b), $W^2$ (c) and $W^2$ (d) of annex problem Eq. (1.5) for a house of cards configuration (a):** for an intermediately high (0.08 μg/mm$^3$) concentration of GO ($D_1$=5.5 x10$^3$ cm$^2$/sec) in water ($D_0$=1.5 x10$^{-5}$ cm$^2$/sec), effective diffusivity computed with Eq. (1.4) is $D_{eff} = \begin{pmatrix} 14 & 2 & 7 \\ 9 & 12 & 4 \\ 6 & 8 & 3 \end{pmatrix} 10^{-5}$ .

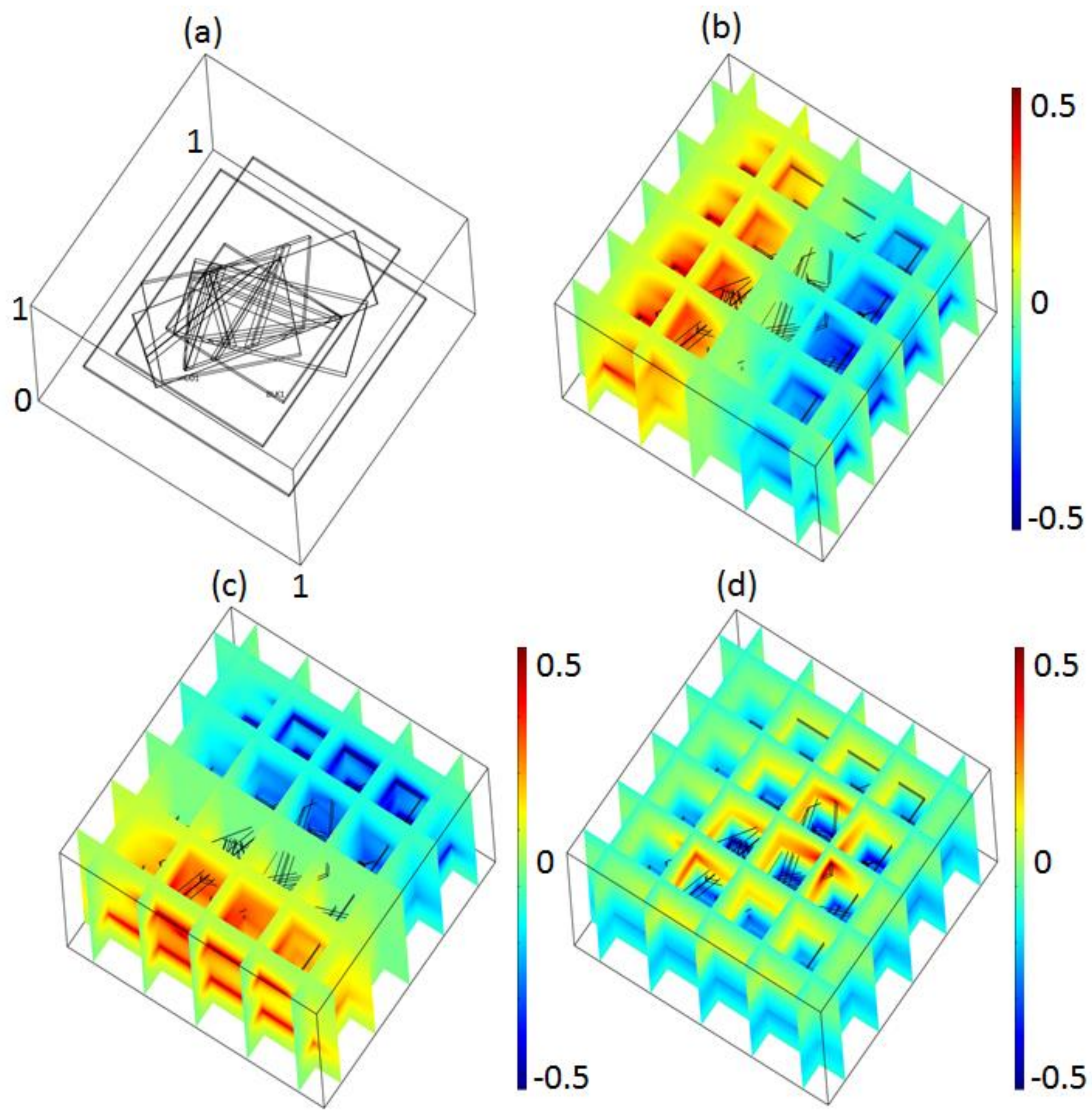

**Supplementary Figure 12: Numerical simulations for low concentration of GO (5.5 $10^3$ $cm^2/sec$) in water (1.5 $10^{-5}$ $cm^2/sec$) in a laminated configuration, in order to calibrate the space and time scales between simulations and experiments.** *(a)* A long water-filled rectangular domain (50 µm in height) with no flux boundary conditions on the left, right and bottom sides; an imposed concentration on the top and GO flakes overlying a porous polyethylene terephthalate (PET) membrane. *(b,d)* Concentration of the peptide at time steps t=1 s and t= 200 s. *(c,e)* Peptide diffusion on low (0.05) and high (0.5) density of GO flakes (in units of $\mu g/mm^3$). *(f,g)* Zoom on low (f) and higher (g) density of GO. Linear color scale ranges from vanishing (dark blue) to high (red) concentration. Here, numerical simulations are reported for a micro-meter lengthscale, and time steps are in microseconds to capture transient effects in drug diffusion delay with an equilibrium state reached in hundredths of seconds, but when we consider millimeter units, typical time scale runs in seconds, and is then converted into minutes for comparison with experiments.

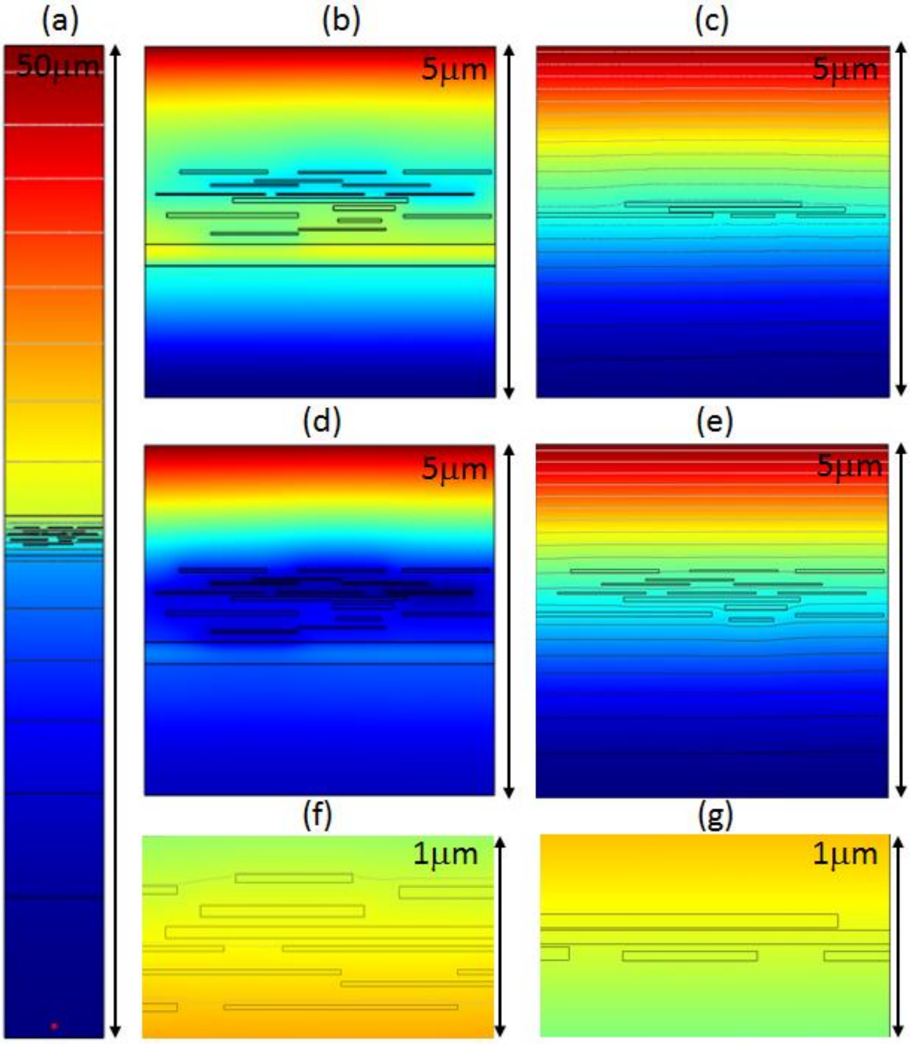

Supplement: Supplementary Methods, Results and Figures [file rsif20170949supp1.pdf]
